# The burden of road traffic accidents in a French Departement: the description of the injuries and recent changes

**DOI:** 10.1186/1471-2458-9-386

**Published:** 2009-10-13

**Authors:** Annabelle Lapostolle, Blandine Gadegbeku, Amina Ndiaye, Emmanuelle Amoros, Mireille Chiron, Alfred Spira, Bernard Laumon

**Affiliations:** 1 INSERM U822, 82 rue du Général Leclerc, Secteur bleu - porte 26, 94276 Le Kremlin Bicêtre, France; 2 INRETS - UMRESTTE, 25, avenue François Mitterrand, 69675 BRON Cedex, France

## Abstract

**Background:**

A significant reduction in road traffic accidents has been observed since prevention measures were introduced by the French public authorities in 2002. The goals of this study are to describe the burden of road traffic accidents in a French Departement, and to identify changes if any between the periods 1997-2001 and 2002-2006 on the basis of the disability adjusted life years (DALY).

**Methods:**

Years of lost life (YLL) and years lived with disability (YLD) were calculated for two periods using the mortality and incidence data in the Rhone Departement Registry of Road Traffic Accident Casualties.

**Results:**

YLD and YLL that are related to road traffic accidents are at their maximum value between 15 and 24 years of age. For men, intracranial fractures and intracranial injuries dominate, and for women it is spinal cord injuries that account for highest rates of YLD. A reduction in the rates of YLL and YLD has been observed for both genders and all age groups between 1997-2001 and 2002-2006.

**Conclusion:**

The reduction in DALY between the two periods is explained both by the reduction in the number of fatalities and injuries but also by an increase in the age at which they occur.

## Background

Disability adjusted life years (DALY) are a summary measure of population health that aims to quantify the proportion of years of life that are lost in a population as a result of early death or disability [1]. Several recent studies [2-4] have highlighted the usefulness of DALY for quantifying the impact of road traffic accidents.

Worldwide, road traffic accidents are estimated to be the 8th cause of DALY, accounting for 2.6% of the total at an estimated annual cost of $518 billion in 2004 [5]. An approximate estimation of the burden of disease in France in 2000-2001 classified road traffic accidents as the fourth cause of DALY among men and the 12th among women [6].

Preventing injuries caused by road traffic accidents has been a major priority of the public authorities for several years. This has led to an increase in monitoring and enforcement measures, in particular by setting up automatic speed radars). From 2002 after these measures were taken, risky driving behaviours, the number of accidents and mortality rates have decreased significantly [7-10]. Between 2002 and 2008, the number of deaths injuries decreased of 44.4% and 31.8% respectively, which correspond to an estimation of 12000 life saved for the 6 years [11].

One of the goals of the French public health law of 2004 was to "achieve a marked and sustained reduction in the number of fatalities and severe secondary consequences resulting from road traffic accidents between now and 2008". At the end of 2007, the head of State set a goal of reducing the annual number of deaths to below 3,000 in 2012 (compared to approximately 5000 in 2006 according to the ONISR ).

The present study aims (i) to use DALY values to describe the traffic accident burden in a French Département in order to quantify their consequences in terms of the public health measure of burden and (ii) to measure and quantify recent changes according to this indicator. For this purpose, we have calculated the DALY from road traffic accidents in the Rhône Département between 2002-2006. At the same time, a comparison with results calculated from a previous period (1997-2001) was performed. This was made possible by the fact that since 1995 a registry of road traffic accident casualties has been held in the Rhône Département [12].

## Methods

### The Rhône Département Road Traffic Accident Casualty Registry

The Rhône Département has a population of 1.6 million. Since 1995 casualties caused by road traffic accidents in the Département have been recorded on a continuous basis in a registry that is recognized by the National Registrys Committee. The criterion for inclusion on the registry is not place of residence but the location of the accident. This decision was made because it is impossible to include residents of the Département who are involved in accidents all over the world, because the main part of road risk occurs near one's residence (in Rhône 1997-2006, 89,2% of victims were living in the département), and because it was necessary to make comparisons with the results obtained by the international accident studies community. Data collection involves all 282 of the healthcare structures which administer care to road traffic casualties in the Rhône Département: Département fire and emergency service,, mobile emergency units (SAMU, SMUR), emergency, treatment of shock, resuscitation, forensic medicine, surgery, rehabilitation and convalescence departments, including those outside the Rhône, who filled in a registry datasheet for each casualty. The recorded health events are death or injury (at least one injury as defined by the Abbreviated Injury Scale (AIS) [13], during a road traffic accident involving at least one moving vehicle (including roller skates and skateboards). Pedestrian falls are thus excluded. The registry includes both inpatients and outpatients, i.e. all casualties, whether hospitalised or not. The casualties and/or their family were contacted by posters or letters and asked to provide certain missing items of data, in particular details about the accident location. For each casualty, the datasheets from the different sources were combined under the same identifier when data was entered into the database. Indeed, for each casualty, injury assessment is based on the whole set of diagnoses provided by the different health services the casualty may have visited. Plain text diagnoses are coded by the registry physician according to the Abbreviated Injury Scale (AIS), 1990 revision. The deceased casualties are registered by mobile emergency units as well as forensic medicine institutes and mortuaries. The cross-checking, coding, checking and data entry had to be performed rigorously and efficiently to achieve optimum data quality: 122,498 datasheets were processed between 1997 and 2006. More information about the registry and data collection have been published elsewhere [12], and its completeness has been assessed [14].

### The Abbreviated Injury Scale

The injuries were described verbally on the basis of medical observations, then coded using the AIS by the Registry doctor. This international classification of traumatic injury includes descriptions of the injury (affected body region, anatomical structure, specific anatomical structure and nature of injury) and an immediate severity level. On the basis of the initial injuries the IIS (Injury Impairment Scale) can be used to assess the nature and severity of likely impairment one year later [15]. The IIS was proposed by Hirsh and Eppinger [16], and completed by States and Viano [17] in work for the AAAM (Association for the Advancement of Automotive Medicine) and makes it possible to evaluate the consequences of injuries at the time they are sustained. The IIS assigns an impairment score to each injury in the AIS, ranging from 0 (no impairment) to 6 (maximum impairment). The IIS values were assigned on the basis of consensus between 35 experts. They take account of mobility, cognitive capacities, aesthetic, sensory or sexual impairment and/or pain.

### The choice of AIS codes

In order to count the casualties in the Registry presenting with each type of injury considered in the Global Burden of Disease (GBD) method [18], a correspondence table was drawn up between the ICD 10 codes attributed to the categories of injury and the AIS codes [see Additional file 1].

In order to identify the short- and long-term consequences, the AIS codes were divided into two categories. Injuries were placed in the "short term" or "long term" line depending on the forecast level of impairment after one year (IIS). For cranial fractures, IIS 1 injuries were assigned to the short term line, and IIS 2 to 4 injuries to the long term line (there is no IIS 0 cranial fracture). IIS 0 femur fractures were assigned to the short term line, IIS 1 femur fractures to the long term (there is no femur fracture with an IIS code higher than 1). Intracranial injuries with an IIS score of 0 (haemorrages) or 1 (internal organs) were classed as short term, and internal organ injuries with an IIS score of between 2 and 6 were classed as long term. IIS 0 eye injuries were classed as short term, and those with IIS scores of 1 and 2 as long term. Burns to less than 20% of the body surface were classed either as short term (IIS 0) or long term (ISS 1 and 2), and those to more than 20% of the body surface were all classed as long term, with IIS codes between 2 and 5. IIS 0 nervous injuries were classed as short term, and IIS 1 to 5 nervous injuries were classed as long term. It should be noted that some AIS codes, which cover injuries of two types, one bone injury and another to an organ, were placed in both GBD classes: an example of this is the medullary injuries associated with a fracture.

### A census of casualties on the basis of the GBD method injury categories

Of the 97,338 casualties listed in the registry between 1997 and 2006, 28,511 (29.3%) were excluded from the study on the grounds that their injuries led to no disability.

For casualties with more than one of the injuries considered in the GBD method, we decided to count all the injuries and place the casualty in all the relevant injury categories. For example, an individual who sustained a fractured femur and radius appears in both injury categories. A total of 15,550 of the casualties have several injuries, and this group accounted for 23% of the casualties with a disability.

In addition, 8,969 casualties (13%) had sustained several different AIS injuries that are grouped together within the same injury category by the GBD method. In this case, the casualty was counted once in each injury category. For example, an individual with a fractured radius and a fractured ulna was counted a single time in the "fractured radius or ulna" category.

### Calculation of the Disability Adjusted Life Years

The DALY combines mortality and morbidity information. For each condition, the Disability Adjusted Life Years (DALY) consist of the sum of the Years of Lost Life (YLL) and the Years Lived with Disability (YLD) [19].



The YLL are the product of the number of deaths observed (N) and the life expectancy in France in 2000-2001 (L) on the basis of the average age at death for each gender and age group:



where *x *is the age and gender category, N is the number of deaths and *L *is the life expectancy

The deaths that are considered in the calculation consist of all the deaths following a road traffic accident that occurred in the Rhône Département during the study period.

The Years Lived with Disability (YLD) are the morbidity component of the DALY. They are a product of the number of incident cases of each injury among surviving casualties (N), the disability weight (DW) which has a value of between 0 and 1 and the average duration of the disability (L).



where *i *is the injury, *x *is the age and gender category, *N *is the number of incident cases, *DW *is the disability weight and *L *is the average duration

For the purposes of this study, it was decided to use the disability weights determined by the WHO on the basis of world expert opinion as used in the original Global burden of disease study. The data on the average duration of disability were estimated by the WHO for the EURO A region to which France belongs (a region of Europe with low infant mortality and high life expectancy) [20]. For the purposes of the GBD, a case incident of trauma is defined as an event that is serious enough to warrant hospitalization or treatment by the emergency services [18]. This definition is compatible with the inclusion criteria for the registry.

### Calculation of values

It is normal practice in road accident studies, in particular in order to make geographical comparisons, to calculate incidence rates by dividing the number of accident casualties in a zone by its population.

The rates of YLD (per 100,000 inhabitants) were calculated by dividing the annual average number of YLD according to gender and age by the average population of the Département for the period in question (i.e. July 1^st ^1999 for the period 1997-2001 and July 1^st ^2004 for the period 2002-2006). The rates of YLL and DALY were calculated in the same way.

In order to compare the rates for the two periods, the rates for 1997-2001 were standardized for gender and age. The standardized rate was defined as the rate that would have been observed in the studied population if it had the same gender and age structure as a reference population. For the period 1997-2001, this standardized rate was calculated by weighting the observed rates for different ages by the age structure at July 1^st ^2004 for each gender in the Rhône Département.

## Results

The most frequent injury categories, irrespective of gender and the studied period, were open wounds, followed by fractures, dislocations and sprains (Table 1). Men sustained the largest number of injuries, for all the injury categories considered, with the exception of dislocations which were more frequent among women.

**Table 1:** Duration, average disability weights and frequencies for each injury and each period of five years (Rhône)

			**Number of injuries Period 1997-2001**				**Number of injuries Period 2002-2006**			
			
	Duration* (years)	Average disability weight*	**Males**		**Females**		**Males**		**Females**	
**Fractures**										
Skull short term	0.107	0.431	184	0.6%	65	0.4%	137	0.5%	40	0.3%
Skull long term	life long	0.361	34	0.1%	12	0.1%	30	0.1%	5	0.0%
Face bones	0.118	0.223	781	2.5%	315	1.9%	532	1.9%	194	1.4%
Vertebral column	0.14	0.266	465	1.5%	223	1.4%	377	1.4%	190	1.4%
Rib or sternum	0.115	0.199	915	2.9%	655	4.1%	875	3.1%	552	4.0%
Pelvis	0.126	0.247	366	1.2%	244	1.5%	293	1.1%	194	1.4%
Clavicle, scapula or humerus	0.112	0.153	1462	4.7%	497	3.1%	1141	4.1%	359	2.6%
Radius or ulna	0.112	0.180	1598	5.2%	682	4.2%	1241	4.5%	546	3.9%
Hand bones	0.07	0.100	1149	3.7%	287	1.8%	874	3.1%	208	1.5%
Femur short term	0.146	0.372	306	1.0%	79	0.5%	268	1.0%	83	0.6%
Femur long term	life long	0.272	220	0.7%	62	0.4%	196	0.7%	36	0.3%
Patella, tibia or fibula	0.096	0.271	1117	3.6%	371	2.3%	860	3.1%	307	2.2%
Ankle	0.099	0.196	468	1.5%	187	1.2%	457	1.6%	160	1.1%
Foot bones	0.073	0.077	532	1.7%	185	1.1%	472	1.7%	145	1.0%
**Injured spinal cord**	life long	0.725	128	0.4%	78	0.5%	89	0.3%	63	0.5%
**Dislocations**										
Shoulder, elbow or hip	0.034	0.074	436	1.4%	98	0.6%	432	1.6%	119	0.9%
other dislocation	0.019	0.074	4658	15.0%	5659	35.0%	5096	18.3%	5534	39.7%
**Sprains**	0.038	0.064	2187	7.0%	899	5.6%	2164	7.8%	787	5.6%
**Intracranial injuries**										
Short-term	0.067	0.359	244	0.8%	84	0.5%	203	0.7%	63	0.5%
Long-term	life long	0.361	264	0.9%	84	0.5%	258	0.9%	70	0.5%
**Internal injuries**	0.038	0.208	891	2.9%	287	1.8%	839	3.0%	267	1.9%
**Open wound**	0.025	0.108	12457	40.1%	5060	31.3%	10803	38.8%	3964	28.4%
**Injury to eyes**										
Short-term	0.019	0.108	83	0.3%	39	0.2%	81	0.3%	37	0.3%
Long-term	life long	0.311	5	0.0%	0	0.0%	0	0.0%	2	0.0%
**Amputations**										
Thumb, finger	life long	0.134	13	0.0%	1	0.0%	12	0.0%	2	0.0%
arm	life long	0.267	6	0.0%	2	0.0%	2	0.0%	1	0.0%
Toe^5^	life long	0.102	3	0.0%	0	0.0%	11	0.0%	5	0.0%
Foot, leg	life long	0.300	14	0.0%	3	0.0%	13	0.0%	2	0.0%
**Crushing**	0.094	0.218	16	0.1%	8	0.0%	32	0.1%	9	0.1%
**Burns**										
less than 20% (short term)	life long	0.158	3	0.0%	0	0.0%	2	0.0%	1	0.0%
Greater than 20%^6 ^(long term)	life long	0.255	2	0.0%	0	0.0%	13	0.0%	2	0.0%
**Injured nerves**										
Long-term	life long	0.069	21	0.1%	2	0.0%	12	0.0%	3	0.0%
**Poisoning**	0.008	0.611	0	0.0%	0	0.0%	0	0.0%	0	0.0%
										
**Total**			**31028**	**100.0%**	**16168**	**100.0%**	**27815**	**100.0%**	**13950**	**100.0%**

* Begg et al. 2002

### The period 2002-2006

If we examine the Disability Adjusted Life Years for these injury categories, the classification is markedly different as the injuries which account for the largest number of YLD were intracranial injuries, which accounted for a total of 691 YLD which is equivalent to a rate of 42 YLD per 100,000 inhabitants (Table 2). Spinal cord injuries accounted for 680 YLD (i.e. a rate of 41 per 100,000 inhabitants) and fractures 654 YLD (40 per 100,000).

**Table 2:** Annuel numbers and rates of Years lived with disability (YLDs) from injuries by age and gender in Rhône (2002-2006)

	**Fractures**			**Injured Spinal cord**			**intracranial injuries**			**Other injuries**		
	
	**Number of YLD**	**Rate per 100 000 inhabitants**	**%**	**Number of YLD**	**Rate per 100 000 inhabitants**	**%**	**Number of YLD**	**Rate per 100 000 inhabitants**	**%**	**Number of YLD**	**Rate per 100 000 inhabitants**	**%**
**Male**												
**0-14**	81	50.2	15.0%	7	4.5	2.0%	81	50.3	15.3%	3	1.6	3.1%
**15-24**	278	232.3	51.4%	112	93.3	31.2%	231	193.1	43.6%	39	32.6	46.2%
**25-34**	100	83.4	18.4%	113	94.6	31.7%	99	82.4	18.6%	17	14.2	20.1%
**35-44**	46	40.7	8.6%	84	73.7	23.6%	57	50.1	10.8%	13	11.7	15.8%
**45-54**	22	22.5	4.1%	24	24.2	6.7%	35	35.3	6.6%	8	8.0	9.4%
**55-64**	9	10.2	1.6%	15	18.5	4.3%	14	16.8	2.6%	3	3.0	3.0%
**65+**	5	5.0	0.9%	2	1.8	0.5%	12	12.8	2.3%	2	2.2	2.4%
***Total***	***541***	***68.3***	***100.0%***	***358***	***45.1***	***100.0%***	***530***	***66.8***	***100.0%***	***84***	***10.7***	***100.0%***
												
**Female**												
**0-14**	44	28.2	38.8%	0	0.0	0.0%	25	16.4	15.8%	6	4.1	22.2%
**15-24**	29	23.2	25.5%	101	81.8	31.5%	67	54.4	41.7%	8	6.3	27.2%
**25-34**	13	10.6	11.6%	119	96.5	37.1%	24	19.4	14.9%	6	4.8	20.8%
**35-44**	12	10.3	10.8%	67	56.8	20.9%	14	12.1	8.9%	1	1.1	4.5%
**45-54**	4	4.1	4.0%	27	24.7	8.3%	13	12.4	8.3%	3	2.4	8.9%
**55-64**	4	5.1	3.9%	5	5.4	1.4%	10	11.3	6.1%	3	3.3	10.1%
**65+**	6	4.4	5.5%	3	1.8	0.8%	7	5.0	4.4%	2	1.3	6.3%
***Total***	***113***	***13.2***	***100.0%***	***322***	***37.5***	***100.0%***	***161***	***18.8***	***100.0%***	***29***	***3.3***	***100.0%***

Together, the other injury categories which accounted for a large proportion of the total number of injuries accounted for only 7 YLD per 100,000.

For every injury category, the YLD rate was higher for men. For men, the injury categories with the highest YLD rates were fractures (68 per 100,000 vs.13 per 100,000 among women) and intracranial injuries (67 per 100,000 vs.19 per 100,000). In contrast, for women, the highest rates resulted from the spinal cord injuries, although the levels were lower than for men (45 per 100,000 for men and 38 per 100,000 for women).

For men, the maximum rate for all injuries was for the 15-24 age group, except for spinal cord injuries (the maximum rate was for the 15-34 age group). Among women the effect of age was much less pronounced and depended on the nature of the injuries: the maximum for fractures was for the first age group (0-14 years), while for spinal cord injuries it was among the 25-34 year-old age group. Only intracranial injuries exhibited a marked peak among 15-24 year-olds, as for men.

For the period 2002-2006, we estimated a total of 2138 Years Lived with Disability, i.e. a rate of 130 per 100,000 (Table 3). The YLD rates were higher for men (191 per 100,000 vs.73 per 100,000 for women) and for both genders the maximum value was for the 15-24 year-old age group (551 per 100,000 for men and 166 per 100,000 for women).

**Table 3:** Annual numbers and rates of Years of life lost (YLLs), Years lived with disability (YLDs) and Disability adjusted life years (DALYs) from injuries by age and gender in Rhône (2002-2006)

	**YLL**			**YLD**			**DALYs**		
			
	**Number**	**Rate per 100 000 inhabitants**	**%**	**Number**	**Rate per 100 000 inhabitants**	**%**	**Number**	**Rate per 100 000 inhabitants**	**%**
**Male**									
**0-14**	75	46.4	2.7%	172	106.7	11.4%	247	153.1	5.8%
**15-24**	1065	890.3	38.9%	660	551.4	43.6%	1725	1441.7	40.5%
**25-34**	656	548.1	23.9%	329	274.6	21.7%	985	822.7	23.1%
**35-44**	413	361.5	15.1%	201	176.1	13.3%	615	537.6	14.4%
**45-54**	286	287.2	10.4%	90	90.0	5.9%	376	377.2	8.8%
**55-64**	124	148.6	4.5%	40	48.5	2.7%	164	197.2	3.9%
**65+**	122	129.0	4.5%	21	21.8	1.4%	143	150.9	3.4%
***Total***	2742	345.9	100.0%	1513	190.9	100.0%	4255	536.8	100.0%
									
**Female**									
**0-14**	74	48.0	9.1%	76	48.7	12.1%	150	96.8	10.4%
**15-24**	212	171.4	26.0%	205	165.7	32.8%	417	337.1	28.9%
**25-34**	127	102.5	15.5%	162	131.3	26.0%	289	233.9	20.1%
**35-44**	123	103.8	15.0%	95	80.3	15.2%	218	184.1	15.1%
**45-54**	102	93.8	12.4%	47	43.6	7.6%	149	137.4	10.3%
**55-64**	79	90.9	9.6%	22	25.1	3.5%	100	116.0	7.0%
**65+**	100	70.8	12.3%	18	12.5	2.8%	118	83.3	8.2%
***Total***	817	95.3	100.0%	625	72.9	100.0%	1442	168.2	100.0%

Over the period 2002-2006, on average 3559 years of life were lost as a result of accidental death on the road in the Rhône Département. The per capita YLL rate was three times higher among men with a rate of 346 YLL per 100,000 vs.95 YLL per 100,000 for women.

For both genders, the maximum per capita YLL rate was for the 15-24 year-old age group with 890 YLL per 100,000 for men and 171 YLL per 100,000 for women. Overall, for both genders, the per capita YLL rate was higher than the per capita YLD rate: 346 vs. 191 per 100,000 for men and 95 vs. 73 YLD per 100,000 for women.

Over the period 2002-2006, we observed an annual average of 5697 Disability Adjusted Life Years which corresponds to a rate of 358 DALY per 100,000 inhabitants (537 DALY per 100,000 for men and 168 DALY per 100,000 for women). Although men had higher rates for all age groups, the difference between the male and female rates were particularly marked between 15 and 34 years, male DALY rates being approximately 4 times higher than female rates. Put another way, the effect of being young is much less marked among women.

The results for the 1997-2001 period, which are not presented here, show the same injury gender and age structure as those for the period 2002-2006.

### Description of change over time

Over the entire 1997-2006 period, the registry contains 1125 fatal casualties of known age and gender. The average annual number of fatalities fell considerably between the two studied periods with an average of 130 annual fatalities in 1997-2001 and 95 in 2002-2006.

The number of casualties also fell between the two periods, from 36,197 in 1997-2001 to 31,308 in 2002-2006. The average per capita number of injuries was similar during the two periods with 1.2 injuries per person in 1997-2001 and 1.3 injuries per person in 2002-2006.

Table 4 shows the standardized DALY, YLL and YLD rates according to injury category for the two studied periods. One can observe a reduction in the YLD rates between the two periods irrespective of the injury category: for example, the YLD rate for both intracranial fractures and intracranial injuries fell from 53 per 100,000 in 1997-2001 to approximately 40 per 100,000 in 2002-2006. The largest reduction was observed for spinal cord injuries which had the highest YLD rate in 1997-2001 i.e. 62 per 100,000. In 2002-2006, the YLD rate for this category was at the same level as intracranial fractures or injuries, i.e. 41 per 100,000. Among women, spinal cord injuries remained the largest category with a rate falling from 51 to 38 per 100,000, followed by intracranial injuries (29 vs. 19) and fractures (26 vs. 13). Among men, the injury category with the highest rates was fractures (83 vs. 68) followed by intracranial injuries (80 vs. 67). The YLD rates for the category of spinal cord injuries fell considerably for men between the two periods: from 73 to 45 per 100,000.

**Table 4:** Annual numbers and standardized rates of YLLs, YLDs and DALYs (per 100,000) from injury categories by age and gender in Rhone

	**1997-2001**			**2002-2006**		
	**N**	rate*	**%**	**N**	**rate**	**%**
**Males**						
**YLD**	1911	251.1	31.6%	1513	190.9	35.6%
*fractures*	*631*	*82.8*	*10.4%*	*541*	*68.3*	*12.7%*
*injured spinal cord*	*559*	*73.4*	*9.2%*	*358*	*45.1*	*8.4%*
*intracranial injuries*	*610*	*80.1*	*10.1%*	*530*	*66.8*	*12.5%*
*other sequelae*	*112*	*14.7*	*1.9%*	*84*	*10.7*	*2.0%*
**YLL**	4141	544.0	68.4%	2742	345.9	64.4%
**DALY**	6052	795.1	100.0%	4255	536.8	100.0%
						
**Females**						
**YLD**	884	107.4	44.5%	625	72.9	43.3%
*fractures*	*210*	*25.5*	*10.6%*	*113*	*13.2*	*7.8%*
*injured spinal cord*	*420*	*51.0*	*21.1%*	*322*	*37.5*	*22.3%*
*intracranial injuries*	*236*	*28.7*	*11.9%*	*161*	*18.8*	*11.2%*
*other sequelae*	*19*	*2.3*	*1.0%*	*29*	*3.3*	*2.0%*
**YLL**	1104	134.3	55.5%	817	95.3	56.7%
**DALY**	1988	241.7	100.0%	1442	168.2	100.0%
						
**Total**						
**YLD**	2795	176.5	34.8%	2138	129.6	37.5%
*fractures*	*841*	*53.1*	*10.5%*	*654*	*39.6*	*11.5%*
*injured spinal cord*	*979*	*61.8*	*12.2%*	*680*	*41.2*	*11.9%*
*intracranial injuries*	*846*	*53.4*	*10.5%*	*691*	*41.9*	*12.1%*
*other sequelae*	*131*	*8.2*	*1.6%*	*113*	*6.9*	*2.0%*
**YLL**	5245	331.2	65.2%	3559	215.7	62.5%
**DALY**	8040	507.7	100.0%	5697	345.3	100.0%

* standard population: Rhône 2002-2006

For all injuries together, between 1997-2001 and 2002-2006, the YLD rate fell from 251 to 191 per 100,000 for men and from 107 to 73 per 100,000 for women, i.e. a reduction of 24% for men and 32% for women.

The YLL rates also fell during the period considered for both genders with a greater reduction for men: from 544 to 346 per 100,000 (36%) and from 134 to 95 per 100,000 (29%) for women.

While both the mortality and morbidity components of the DALY values fell between the two periods, the greatest reduction was in the YLL rates.

**Figure 1:**
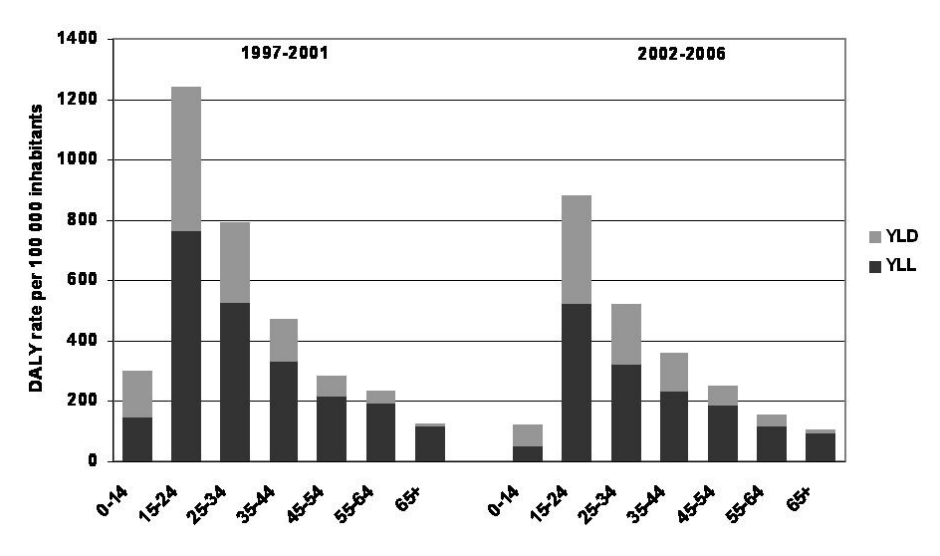
**Annual rates of DALY per 100 000 inhabitants depending on the period and the age, Rhône**.

Over the two periods, the maximum rate of DALY occurred between 15 and 24 years of age, and the rates gradually decreased with age in a similar way in both periods (Figure 1).

A reduction in the YLD, YLL and therefore the DALY rates has been observed for all age groups and both genders. This reduction is the greatest for the youngest age groups. The greatest reduction was observed for 0-14 year-olds, whose rate of DALY was halved between the two periods: 300 per 100,000 in 1997-2001 and 126 per 100,000 in 2002-2006. The rates of DALY fell from 1241 to 880 per 100,000 for the 15-24 year-old age group and from 794 to 523 per 100,000 for the 25-34 year-old age group.

## Discussion

This study set out to assess the impact of road traffic accidents in a French département, the Rhône, using an indicator that combines mortality and morbidity. The findings set out here highlight the high rates of YLL and YLD for young people, particularly in the 15-24 year-old age group.

The data from the registry were used to develop the components from which the estimates of DALYs were constructed. The YLD resulting from a road traffic accident have been broken down on the basis of injury category and type of disability - temporary or long term.

These results highlight gender differences. Rates of YLL and YLD are higher among men for all age groups, but there are also structural gender differences in the type of road traffic accident injuries. Spinal cord injuries are the major category of injury for YLD among women (rate of YLD of 38 p.100 000 casualties), while among men the YLD are caused by all the major injury categories: fractures (essentially long-term cranium and femur fractures), spinal cord injuries and intracranial injuries.

These results highlight the improvement between the two periods with an overall reduction in the rates of YLD (from 177 per 100,000 inhabitants in 1997-2001 to 130 per 100,000 inhabitants in 2002-2006) and above all the rates of YLL (from 331 to 216 per 100,000). This reduction is more marked among children and young adults.

The results obtained for the Rhône département in 2002-2006 are slightly higher than those in other recent studies in European Union countries. Thus, a comparative study of 6 European countries (Austria, Denmark, Ireland, the Netherlands, Norway and the United Kingdom) reports between 200 and 500 DALY per 100,000 for men (Rhône 536 per 100,000) and between 52 and 150 DALY per 100,000 for women (Rhône 168 per 100,000) [2]. A study conducted in the Utrecht region of the Netherlands for 1999-2000 reported 1.2 YLD per 1,000, 2.7 YLL per 1,000 and 3.9 DALY per 1,000 [3] vs., respectively, 1.3 YLD per 1,000, 2.3 YLL per 1,000, and 3.6 DALY per 1,000 in the Rhône. These results concur with the road traffic accident mortality rates in the European Union with a rate of 75 per 1,000,000 for France, which is higher than those for Denmark (56 per 1,000,000), the Netherlands (45) and the United Kingdom (55) in 2006 (EU road accidents database CARE).

As the Rhône is a highly urbanized département, the road traffic accidents which occur there are on average less severe than in France as a whole. Thus, the incident rate for 30 day fatalities was 49 per million inhabitants in the Rhône vs. 91 for France as a whole in 2005 (Observatoire national interministériel de la sécurité routière http://www2.securiteroutiere.gouv.fr/infos-ref/observatoire/index.html). Likewise, the incidence of casualties with severe consequences (IIS 3+) was estimated by the Registry at 5.4 per 100 000 in the Rhône for the period 1996-2003 [21], but at 12.6 per 100,000 for France as a whole over the period 1996-2004 [22]. The difference between the figure for France and the Rhône does not involve multiplication by the same factor in all cases: thus, the incidence of ISS 3+ consequences is, for men, 8.1 in the Rhône vs. 20.6 per 100,000 in France and, for women, 2.9 per 100,000 in the Rhône vs. 5.1 per 100,000 in France. The differences in the scale of incidences are attenuated when injuries of all severities are taken together: 582 per 100,000 for the Rhône vs. 871 per 100,000 for France, in the period 1996-2004. This means that the rates of DALY estimated from the Rhône Registry constitute a lower bound for the rates of DALY for the whole of France.

The durations considered for the calculations in this study are those that were estimated for the GBD method. For long-term disabilities, it has been assumed that individuals are affected for the remainder of their life (life expectancy). However, for some injuries (fractured skull, injured spinal cord, fractured femur and intracranial injury), we have not considered the relative excess risk of dying, which reduces life expectancy [20]. Delayed deaths are therefore not included in the total number of YLL and will be ascribed to a secondary cause (a complication or another condition). We can therefore assume that for these injuries the number of YLL has been underestimated.

On the other hand, casualties who have sustained spinal cord injuries for which only the long term disabilities are considered in the GBD method will perhaps not all be permanently disabled, as the AIS codes used for this category include injuries resulting in transient disabilities. The ICD codes do not permit this distinction with regard to severity to be made. All the AIS codes that correspond to each ICD code were therefore used, leading perhaps to an overestimation of the duration of disabilities, and hence the DALY for spinal cord injuries. This methodological point may have led to an underestimation of the differences between males and females for DALY for spinal cord injuries, as one frequently notices that when the AIS classification is used, the spinal cord injuries of men are more severe than those of women, and more frequently responsible for permanent consequences.

Our decision to use an approach based on injury categories rather than casualties may have led to an overestimation of the results in our study, as the same casualty is ascribed YLD for each of his/her injuries. We nevertheless considered that this approach was preferable to considering only the most severe injury in the calculation. This choice is consistent with other burden of disease studies where a given individual may suffer from different conditions, and therefore be counted twice. On the other hand, the fact that several AIS injuries to the same location are counted once for each injury category may have led to an underestimation of the YLD. Nevertheless, the percentage of casualties falling into this last category was low and the disability resulting from several injuries to the same location can be considered to be equivalent to the disability resulting from a single injury.

For the temporal comparison, the same disability weights and durations for each injury were used for both study periods. The same method was used as in the only other study which deals with temporal changes in DALY [23]. To our knowledge, no study has examined change in disability weights or long term durations. Nevertheless, if we make the hypothesis that the medical care given to crash casualties improved between the two periods, the disability weights and durations would be overestimated in the second period, and the observed reduction between the two periods would be greater than we observed. This hypothesis does not seem to apply to our study in that the studied periods are very close together and no difference in the care administered between the two periods has been observed.

The results we obtained are certainly underestimated for the Rhône département and the observed rates are an even greater underestimation of the situation in the whole of France.

## Conclusion

This study has shown there was a reduction in the rates of DALY for road traffic accidents between the two periods 1997-2001 and 2002-2006 in the Rhône département. This reduction is due to a dual process of a reduction in incidences and an increase in the age at which the events occurred, for both disability and mortality. To conclude, DALY values are an useful means of quantifying mortality and morbidity on the basis of injury categories, ages and genders as well as studying temporal changes.

## Competing interests

The authors declare that they have no competing interests.

## Authors' contributions

AL performed the calculation of DALYs and drafted the manuscript. AS involved in the calculation of DALYs and correction of manuscript. MC, BG, AN, EA, MC and BL participated in the data collection and the redaction of the manuscript, and carried out AIS/ICD correspondence table. All authors read and approved the final manuscript.

## Pre-publication history

The pre-publication history for this paper can be accessed here:



## Supplementary Material

Additional file 1 **Correspondence table between the ICD 10 codes attributed to the categories of injury and the AIS codes**. This table presents the corresponding codes between ICD10 and the AIS for each category of injuries. Click here for file
