# Supplementary material for: The burden of road traffic accidents in a French Departement: the description of the injuries and recent changes
Source: BMC Public Health. 2009 Oct 13;9:386. doi: 10.1186/1471-2458-9-386 (PMC2774692; doi:10.1186/1471-2458-9-386)
Supplement: Additional file 1 — Correspondence table between the ICD 10 codes attributed to the categories of injury and the AIS codes. This table presents the corresponding codes between ICD10 and the AIS for each category of injuries. [file 1471-2458-9-386-S1.DOC]

| ***Nature of injury category*** | ***ICD 10 codes*** | ***AIS90 codes*** |
| --- | --- | --- |
| **Fractures** |  |  |
| Skull short term | S02.0/1/7/9,T90.2 | 1 5 00 00/1 5 02 00 to 1 5 02 04/ 1 5 04 00/1 5 04 02 |
| Skull long term | S02.0/1/7/9,T90.2 | 1 5 02 06/1 5 04 04 to 1 5 04 08 |
| Face bones | S02.2/6/8 | 2 5 02 to 2 5 12/2 5 18 00 |
| Vertebral column | S12,S22.0/1,S32.0/7,T91.1 | 3 4 02 08/3 4 02 10/3 5 02 00  4 4 26 12 to4 4 26 16  6 3 06 06/6 3 06 10/6 3 06 24/6 3 06 28/6 3 06 34/6 3 06 38  6 4 02 04/6 4 02 08/6 4 02 14/6 4 02 18/6 4 02 24/6 4 02 28/6 4 02 46/6 4 02 50/6 4 02 64/6 4 02 68  6 4 04 04/6 4 04 08/6 4 04 14/6 4 04 18/6 4 04 24/6 4 04 28/6 4 04 46/6 4 04 50/6 4 04 64/6 4 04 68  6 40 06 04/6 40 06 08/6 40 06 14/6 40 06 18/6 40 06 24/6 40 06 28/6 40 06 46/6 40 06 50/6 40 06 64/6 40 06 68  6 5 02 16 to 6 5 02 34/6 5 04 16 to 6 5 04 34/6 5 06 16 to 6 5 06 34 |
| Rib or sternum | S22.2-9 | 4 5 02 10 to 4 5 02 68/4 5 08 02/4 5 08 04 |
| Pelvis | S32.1-5/8,T91.2 | 8 5 26 00 to 8 5 26 10/8 5 28 00/8 5 30 00 |
| Clavicle, scapula or humerus | S42,S49.7 | 7 5 16 00/7 5 18 00/7 5 22 00/7 5 26 00 to 7 5 26 06/7 5 30 00 |
| Radius or ulna | S52,S59.7,T10,T92.1 | 7 5 28 00 to 7 5 28 06/7 5 32 00 to 7 5 32 06/ |
| Hand bones | S62,S69.7,T92.2 | 7 5 20 00 to 7 5 20 04/7 5 24 04 |
| Femur short term | S72,S79.7 | 8 5 18 00/8 5 18 04 to 8 5 18 20 /8 5 18 16 |
| Femur long term | S72,S79.7 | 8 5 18 01/8 5 18 02/8 5 18 22/8 5 18 24 |
| Patella, tibia or fibula | S82.0-4,S82.7/9,S89.7,T12 | 8 5 16 06/8 5 24 00/58 5 34 04 04 to 8 5 34 10/8 5 34 20/8 5 34 22 |
| Ankle | S82.5-6/8 | 8 5 16 08 to 8 5 16 12/8 5 34 12 to 8 5 34 18 |
| Foot bones | S92,S99.7 | 8 5 14 00/8 5 20 00/8 5 32 00/8 5 36 02/8 5 32 06 |
| **Injured spinal cord** | S14,S24,S34,T06.0/1,T08,T91.3 | 3 3 02 99/3 3 04 99/6 3 02 02 to 6 3 02 14/6 3 02 16 to 6 3 02 26/6 3 02 99/6 4 02 00 to 6 4 02 28/ 6 4 02 40 to 6 4 02 68/6 4 04 00 to 6 4 04 68/ 6 4 06 00 to 6 4 06 68/  6 3 02 99/6 3 02 10 to 6 3 02 26/6 3 02 02 to 6 3 02 08/6 3 04 02 to 6 3 04 14/ 6 3 06 00 to 6 3 06 38/3 3 02 99/3 3 04 99 |
| **dislocations** |  |  |
| Shoulder, elbow or hip* | S43,S73 | 7 5 10 30/7 5 10 /8 5 6 10 to 18 5 6 8 7 5 2 20/7 5 2 30 /7 5 12 20/7 5 12 30 |
| other dislocation | S03.0-3, S13, S23, S33, S53, S63.0/1, S83.1-3, S93.1-3, T03, T11.2, T13.2, T14.3, T92.3, T93.3 | 2 5 16 04/2 5 14 02/6 5 0 2 03/6 5 02 04 to 6 5 02 12 /6 4 02 78/6 4 04 78/6 4 06 78/6 5 04 03 to 6 5 04 12/6 5 06 03 to 6 5 06 12/7 5 06 20/7 5 06 30/7 5 14 30/7 5 04 04/8 5 8 06 to 8 5 8 18/8 5 10 06 to 8 5 10 14/8 5 12 04 /8 5 12 06 |
| **Sprains** | S03.4/5,S16,S29.0,S39.0,S46,S56,S63.5-7,S66,S76,S83.4/7,S86,S93.4/6,S96,T06.4,T11.5,T13.5,T14.6,T92.5,T93.5 | 2 5 16 02/4 5 02 99/4 5 02 02/7 4 2 20/7 4 02 00/7 4 02 10/7 4 04 00/7 4 06 00/7 5 10 40/7 5 14 20/7 5 04 02/8 4 06 00/8 4 08 02/8 4 08 04/8 4 4 04/8 5 8 22/8 5 8 26/8 4 02 00 to 8 4 02 04/8 4 04 02/8 5 02 06/8 4 04 04 /8 4 04 06/8 5 10 02/8 5 12 02 |
| **Intracranial injuries** |  |  |
| Short-term | S06,T90.5 | 1 4 04 14/1 4 04 18/1 4 04 50/1 4 04 54/1 4 04 62/1 4 04 66/1 4 04 70  1 4 06 30/1 4 06 32/1 4 06 60/1 4 06 62/1 4 06 68/1 4 06 70/1 4 06 82 to 1 4 06 86/1 2 02 99 to 1 2 28 06 except codes beginning by 1 or 2 in “long terme” |
| Long-term | S06,T90.5 | 1 4 2 99/1 4 02 02 to 1 4 02 10/1 4 04 99/1 4 04 02 to 1 4 04 10/1 4 04 22 to 1 4 04 46/1 4 04 58/1 4 04 74/1 4 04 78/1 4 06 99/1 4 06 02 to 1 4 06 29/ 1 4 06 34 to 1 4 06 56/ 1 4 06 64/1 4 06 66/1 4 06 72 to 1 4 06 80/1 4 06 88/1 4 06 90/1 4 07 99/1 2 02 04/1 2 04 04/1 2 06 02/1 2 10 04/1 2 14 04/1 2 18 04 |
| **Internal injuries** | S25-S27,S35-S37,S39.6,T06.4,T91.4/5 | 4 2 all/4 4 10 all/4 4 12 00/4 4 13 00/4 4 16 all/4 4 02 all/4 4 04 00/4 4 06 all/4 4 08 all//4 4 14 all//4 4 18 all//4 4 22 all/4 4 24 02/4 4 26 all/4 5 02 14/4 5 02 22/4 5 02 32/4 5 02 42/4 5 02 52/4 5 02 64/5 2 all/5 4 04 all/5 4 08 all/5 4 10 all/5 4 12 all/5 4 14 all/5 4 18 all/5 4 20 all/5 4 22 all/5 4 28 all/5 4 36 all/5 4 38 all/5 4 42 all/5 4 44 all/5 4 02 all/5 4 06 all/5 4 16 all/5 4 24 all/5 4 26 all/5 4 32 all/5 4 34 all/5 4 48 all/5 4 50 all/5 4 52 all |
| **Open wound** | S01,S08,S11,S15,S21,S31,S41,S45,S51,S55,S61,S65,S71,S75,S81,S85,S91,S95,T01,T11.1/4,T13.5,T14.6,T90.1,T92.5,T93.5 | 1 1 60 all/1 1 00 all/1 1 02 all/1 1 04 all/1 1 06 all/1 1 08 all/2 1 60 all/2 1 00 all/2 1 02 all/2 1 04 all/2 1 06 all/2 1 08 all/3 1 60 all/3 1 00 all/3 1 02 all/3 1 04 all/3 1 06 all/3 1 08 all/3 4 02 99/3 4 02 02 to 3 4 02 10/3 4 06 04 to 3 4 06 08/3 2 all/4 1 10 00/4 1 60 all/4 1 00 02 to 4 1 00 08/5 1 60 all/5 1 00 all/5 1 02 all/5 1 04 all/5 1 06 all/5 1 08 all/5 4 30 20 to 5 4 30 24/5 4 46 20 to 5 4 46 24/5 4 54 20 to 5 4 54 24/5 4 56 20 to 5 4 56 24/ 7 1 60 all/7 1 00 all/7 1 02 all/7 1 04 all/7 1 06 all/7 1 08 all/ 7 2 all/ 8 1 60 all/8 1 00 all/8 1 02 all/8 1 04 all/8 1 06 all/ 81 08 all/8 2 all/9 1 06 00 |
| **Injury to eyes** |  |  |
| Short-term | S05,T90.4 | 2 4 4 99/2 4 4 08 to 2 4 4 16/2 4 06 all/2 4 08 00/2 4 10 00/2 4 12 00/2 4 14 99/2 4 16 99 |
| Long-term | S05,T90.4 | 2 4 04 02/2 4 10 02/2 4 12 02 |
| **Amputations** |  |  |
| Thumb | S68.0 | 7 5 24 02 |
| finger | S68.1/2 |
| arm | S48,S58,S68.3-9,T05.0/2,T11.6 | 7 1 10 00 |
| Toe^5^ | S98.1/2 | 8 5 36 04 |
| foot | S98.0/3/4,T05.3 | 8 1 10 all |
| leg | S78,S88,T05.4/6,T13.6 |
| **Crushing** | S07,S17,S28,S38,S47,S57,S67,S77,S87,S97,T04,T14.7,T92.6,T93.6 | 3 4 02 12/3 4 06 10/4 4 26 16/5 4 30 26/5 4 40 24/5 4 46 24/5 4 54 26/5 4 56 26/8 5 36 06/8 1 30 00 |
| **Burns** |  |  |
| less than 20% short term | T31.0/1 | 9 1 20 06/9 1 20 08 |
| Less than 20% long term | T31.0/1 | 9 1 20 12 to 9 1 20 16 |
| 20 to 60% short term | T331.2/5 |  |
| Greater than 60% short term | T31.6/9 |  |
| 20 to 60% long term | T331.2/5 | 9 1 20 18 to 9 1 20 30 |
| Greater than 60%^6^ long term | T31.6/9 |
| **Injured nerves** |  |  |
| Short-term | S04,S44,S54,S64,S74,S84,S94,T06.2,T11.3,T13.3,T14.4 | 1 3 02 02/1 3 04 99/1 3 04 02/1 08 99/1 3 08 02/1 3 10 99/1 3 10 02/1 3 12 all/1 3 14 99/1 3 14 02/1 3 16 99/1 3 16 02/1 3 20 all/1 3 22 99/1 3 22 02/1 3 24 99/1 3 24 02/1 3 26 99/1 3 26 02/7 3 02 all/7 3 04 99/7 3 04 10/8 3 02 all/8 3 06 02 |
| Long-term | S04,S44,S54,S64,S74,S84,S94,T06.2,T11.3,T13.3,T14.4 | 1 3 02 99/1 3 02 04/1 3 04 04/1 3 06 all/1 3 08 04/1 3 10 04/1 3 14 04 /1 3 16 04/1 3 18 all/1 3 22 04/1 3 24 04/1 3 26 04/7 3 04 20 to 7 3 04 50/8 3 04 all/8 3 06 99/8 3 06 04 to8 3 06 10/ |
| **Poisoning** | T36-T65,T96-T97 | 9 1 92 all |
